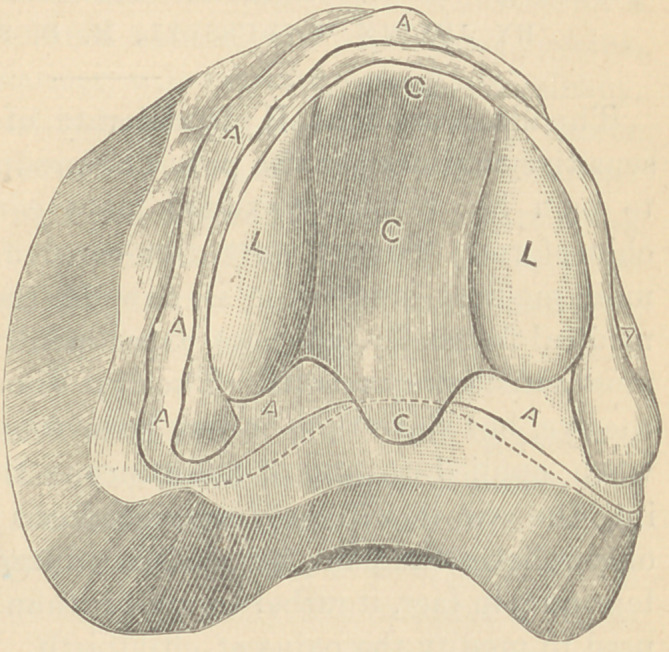# Adapting Artificial Dentures

**Published:** 1885-01

**Authors:** C. H. Land

**Affiliations:** Detroit, Mich.


### ADAPTING ARTIFICIAL DENTURES.

BY DR. C. H. LAND, DETROIT, MICH.

In the fitting of artificial teeth to the various conditions of the mouth, there seems to be much confusion. Every man has a way of his own, and will take pride in telling you of the remarkable degree of success he has attained in certain difficult cases; how all his fellow-practitioners have failed, and how he in some profound and mysterious way succeeded. Then there is the individual who will bore you with an hour's lecture on how to take a wax impression. If you show him some slight improvement, he will claim priority of invention by twenty years. He always makes a fit, never fails, and his patients can with perfect ease eat green corn off the cob. Then we have the plaster impression man, the modelling compound man, the air chamber man, the suction plate man, the no air chamber man, and the man who talks about the cohesion of the plate. In the preparing of metallic dies one man will claim that zinc is the best; another declares it not proper for the purpose, and that Babbitt's metal is the only thing fit to use, and these men, with all their apparent wisdom, will hesitate about coating a plastic impression with sandarach varnish $\frac{1}{1000}$ of an inch in thickness, for fear of spoiling the adaptation.

However, a careful investigation will show that about the same results can be obtained from several directions, proving the fault to be with the men, and not in the material. In order to comprehend the principles involved we must progress scientifically, search out the real facts, and see if every part can be measured mathematically. When this is done ultimate truths will be revealed, and the fitting of artificial dentures will be made easy. No one can reach the maximum of success unless he becomes thoroughly familiar with every condition of the mouth. There is the rigid, the soft, the large, the flat, the angular, and the dry, or it may be three of these conditions in one. Each will represent a different degree of contact and retaining force, according to the size and different angles, and whether they are moist or dry, hard or soft. A small, dry, flat and rigid mouth cannot be made to support much more than the actual weight of the denture, and will represent the minimum retaining force; say about two ounces. On the contrary, a large, soft and moist mouth will conveniently support several pounds. In preparing a denture so as to secure the most perfect adaptation, capillary attraction is the force to be relied on, and every effort that will have a tendency to augment or increase it will be in the right direction. It causes the denture to adhere with a force of about two pounds to the square inch. On the contrary, with an air space and the exhausting power of the tongue, the retaining force is reduced to about two ounces to the square inch, which would be one ounce for the usual small air-chamber. From this it will be seen that capillary attraction represents a retaining force thirty times greater. In order to secure the best results, four-fifths of the central portion of the denture must be converted into a partial vacuum, about the sixtieth part of an inch in depth, as a temporary means of establishing an adaptation by capillary force. Experience has demonstrated that, eventually, all air spaces become filled with the tissues, proving that a permanent partial vacuum cannot be maintained; and yet, if it were possible, the utility would be of less importance than capillary attraction. When four-fifths of the lingual surface is relieved, the denture is prevented from coming in contact with the central portion of the dental arch, causing the outer margin to become adapted to the alveolar ridge first. This prevents the plate from rocking or riding on the rigid portion of the palatine arch, and eventually secures the maximum contact over the entire surface. The mouth, being of a yielding nature, soon conforms to this relief, producing a balanced contact, and a denture that is maintained entirely by capillary attraction. If the exhausting power of the tongue were strong enough, and the tissues of the mouth would tolerate an atmospheric pressure of only one pound to the square inch, no better means could be devised for maintaining artificial teeth in position. It differs from capillary attraction by offering a resistance in every direction, while the latter, when pushed sideways, slips or slides, the intermediate fluid causing the two surfaces to roll or slide past each other. This will account for the great difficulty in fitting angular mouths. A narrow and high arch presents so little horizontal surface as to reduce the retaining force to its minimum. Capillary attraction is greatest when the exertion is in a direct angle from a horizontal surface, and in order to obtain the best results in fitting angular mouths we must resort to friction by trimming all the perpendicular angles of the cast, thus forcing the mouth into a smaller space than the impression represented. In applying these directions to the various shaped mouths, they will bear a considerable modification. A mouth that is flat and rigid needs the least possible change made in the denture. A very slight relief, gradually increasing toward the center, will bring about the best results, and in this case the man who is guided simply by a good impression is almost as successful as the one who makes the relief. On the contrary, a mouth in which the alveolar ridge is soft and the central portion of the dental arch very rigid, unless considerable relief be made, the denture will find its first contact on the palatine surface, and close adaptation will be destroyed. By relieving four-fifths of the central portion, first contact is thrown to the outside, and maximum adaptation is secured. Any rigid portion of the mouth coming in direct contact with a rigid denture will destroy a perfect adaptation. These parts are usually found in the central portion of the palatine surface, and the dense parts of the maxillary bone, and unless some relief is given the denture will rock or ride, either forward backward, or sideways. By reference to the following illustrations it will be seen that L L C C C mark the position of a lead or tin matrix to form a proper relief that will be effective in all cases where the alveolar ridge is soft and spongy, and the central portion of the palatine surface rigid as far back as the soft palate. The lobes, L L, are designed to give additional relief to the dense parts of the maxillary bone, and are about the thirty-second part of an inch in thickness, with edges beveled so as to avoid any acute angles. The central portion, C C C, is about the sixtieth part of an inch thick. The dotted lines indicate the extent of the denture, showing that when completed there will be a space or opening at C. This serves as a complete relief, and permits the air to pass between the denture and the mouth. In order to get an immediate suction it will be necessary to insert a moist piece of cotton at this particular point, until the mouth so conforms to the denture as to close the space, and this will take place in from three to four days. The letters A A A A A indicate the parts of the mouth that, when soft and spongy, will bear increased pressure, and this is brought about by trimming the plaster cast just in proportion to the conditions found in each case. If rigid, no trimming will be tolerated.

Finally, in the construction of artificial dentures, the first step is a correct impression; second, a proper diagnosis of the conditions presented in each case; third, a proper trimming of the plaster cast for relief. Then keeping in view the fact that the mouth will readily conform to the denture, and that capillary attraction is of far more value than atmospheric pressure, it is possible to reach the utmost degree of success.

## Figures and Tables

**Figure f1:**